# Health, functioning and social engagement among older people living in long-term care facilities during the COVID-19 lockdown in Finland: a register-based cohort study

**DOI:** 10.1186/s12889-025-22032-8

**Published:** 2025-03-08

**Authors:** Johanna Edgren, Jokke Häsä, Mari Aaltonen

**Affiliations:** 1 Department of Healthcare and Social Welfare, Finnish Institute for Health and Welfare, Helsinki, Finland; 2 Department of Data Management of Healthcare and Social Welfare, Finnish Institute for Health and Welfare, Helsinki, Finland; 3 Gerontology Research Center, Tampere University, Tampere, Finland

**Keywords:** COVID-19 lockdown, Functioning, Health, Long-term care facilities, Older people

## Abstract

**Background:**

There is a lack of consistent evidence on the effects of the COVID-19 lockdown among older long-term care facility (LTCF) residents. We utilised a versatile and comprehensive register-based data to assess the impact of the lockdown and to explore what kinds of individual-level factors were associated with changes in functioning and wellbeing of the older LTCF residents during the lockdown in 2020.

**Methods:**

This retrospective register-based cohort study (*n* = 7 260) with a 6-month follow-up utilised Resident Assessment Instrument (RAI) data combined with data on confirmed COVID-19 infections and death records of LTCF residents aged 65-year-old and older. Logistic regression analyses were conducted to detect cohort effects on health stability, cognitive performance, coping with activities of daily living (ADL), and social engagement. Additional subgroup analyses were performed to explore the effect among the oldest (85 years old and older), most severely cognitively impaired individuals (dementia diagnosis and Cognitive Performance Scale score 4–6), and those who experienced the lowest social engagement (Social Engagement Scale score 0–1) at baseline.

**Results:**

When all the RAI assessed LTCF residents were included in the analyses, belonging to the lockdown cohort was not observably associated with a decline in health stability, cognitive performance, coping with ADL, or social engagement. According to the subgroup analyses, the health stability of the oldest residents and the cognition of the most severely cognitively impaired residents deteriorated more in the lockdown than in the comparison cohort.

**Conclusions:**

The COVID-19 lockdown was not observably associated with deterioration in health, cognitive or ADL functioning, or social engagement among Finnish LTCF residents. However, subgroup analyses suggested that the effects of the lockdown were the most detrimental among the most severely cognitively impaired and the oldest residents. The vulnerability between different subgroups should be considered more closely in exceptional circumstances due to infectious diseases in the future and provide deliberately older people the opportunity to experience the physical closeness of their loved ones despite possible infections.

**Supplementary Information:**

10.1186/s12889-025-22032-8

## Introduction

The COVID-19 epidemic affected people’s lives extensively, particularly during the first wave in 2020. The lives of frail older people living in long-term care facilities (LTCF) were particularly affected since there was early evidence that the mortality rate of COVID-19 was greater and the health impact more severe among older people than the general population [11, 15, 44, 45]. In Finland, the Emergency Powers Act came into force on 17 March 2020. Accordingly, people’s fundamental rights were restricted, and health care resources were secured by specific actions to prevent the spread of the virus and to protect people from its detrimental effects [13]. In this research, we focus solely exploring the impact of the COVID-19 lockdown on older LTCF residents by applying age matched cohort design and utilising versatile national level register-based data including information on health, functioning, well-being, confirmed COVID-19 infections, and time of death.

In Finland, people aged 70-year-old and older and home care workers who visited older people were given targeted instructions on how to protect themselves from them from the virus and how to prevent the virus from spreading during the first wave of COVID-19  [13]: Older people were advised to refrain from contact with other persons and stay at home in quarantine-like conditions: family and friends were advised to avoid any non-essential visits to anyone over 70  [13]. Also, careful hand and cough hygiene, and using protective equipment such as disposable protective gloves and surgical mouth-nose protection were required. However, the most detrimental social distancing occurred in LTCF of older people as the LTCF were “locked down” for several months in 2020 in addition to the general instructions and restrictions described above [13]. Anyhow, the evidence on the effects of the COVID-19 lockdown period on health, well-being, and functioning of the LTCF residents remains conflicting and inconsistent: For instance, in Finland most of the earlier studies exploring the impact of the COVID-19 lockdown among older people have focused on home-dwelling population as it was difficult, or even impossible, to access LTCF and reach to the older LTCF residents. Additionally, majority of the earlier research describing the impact of the COVID-19 lockdown on the Finnish LTCF residents have not been representative due to data or methods that have been available and utilised by researchers. Moreover, it is vitally important to study the impact of the COVID-19 lockdown among different populations as they may respond to exceptional circumstances in various manners: For instance, the Nordic and Finnish population are unique in a distinctive manner since the history of severe pandemics, wars, and related famine as well as the harsh climate, difficult terrain, and socially restrained culture.

Although home care is the primary form of service for older people in Finland, approximately 3.6% of older people aged 65-year-old and older are living in LTCF [30]. Round-the-clock care in LTCF is considered when living at home is not possible even with intensive measures of care and support [29]. During the first wave of COVID-19, the LTCF were locked down to all visitors, including family and friends, as well as therapists and other health care professionals coming from outside of the unit. Additionally, the use of common spaces in the units was strictly restricted to avoid all social contact. Thus, formal, and informal social activities, rehabilitation, therapies, and special treatments were put on hold, exposing the residents to a potential deterioration in health and functioning. Additionally, the use of personal protective equipment, such as a face mask and gloves, was required from health care professionals and visitors when visits to the LTCF residents were allowed, which may have confused the residents, especially those with cognitive decline [14].

The goal of the visiting restrictions in LTCF was to prevent the spread of the virus and hence to protect older people. However, it has been proposed that restrictions may have had an even more detrimental effect than the virus itself on the well-being and functioning of older residents [35, 42]. Additionally, the changes and restrictions may have been very confusing for residents with impaired cognition or diagnosed dementia [14]. According to some earlier research, the lockdown resulted in the deterioration of health, well-being and functioning among older people living in LTCF [9, 25, 35]. In addition, residents and family members have experienced anxiety, grief, and severe stress (e.g., [35]). However, some large register-based studies have demonstrated opposite findings such as lockdown did not affect significantly on psychosocial well-being of the older LTCF residents (e.g., [27]).

The aim of this study was to assess functioning and well-being among older people living in LTCF during the COVID-19 lockdown in Finland using comprehensive national level register data drawn from the Resident Assessment Instrument (RAI) combined with date of death and confirmed COVID-19 infections. Because physical and cognitive functioning, as well as individual service needs of the LTCF residents can be diverse and are typically age related, we studied the effects of COVID-19 lockdown separately among those with severe cognitive decline and among the oldest residents aged 85 years old and older. The detailed research questions were: What was the impact of the COVID-19 lockdown on the deterioration of health stability, functioning, cognition, and social well-being among older residents living in LTCF? What kinds of individual factors were associated with the deterioration of health stability, social engagement, cognitive performance, and coping with activities of daily living during the COVID-19 lockdown among older residents living in LTCF?

### Methods

**Fig. 1:**
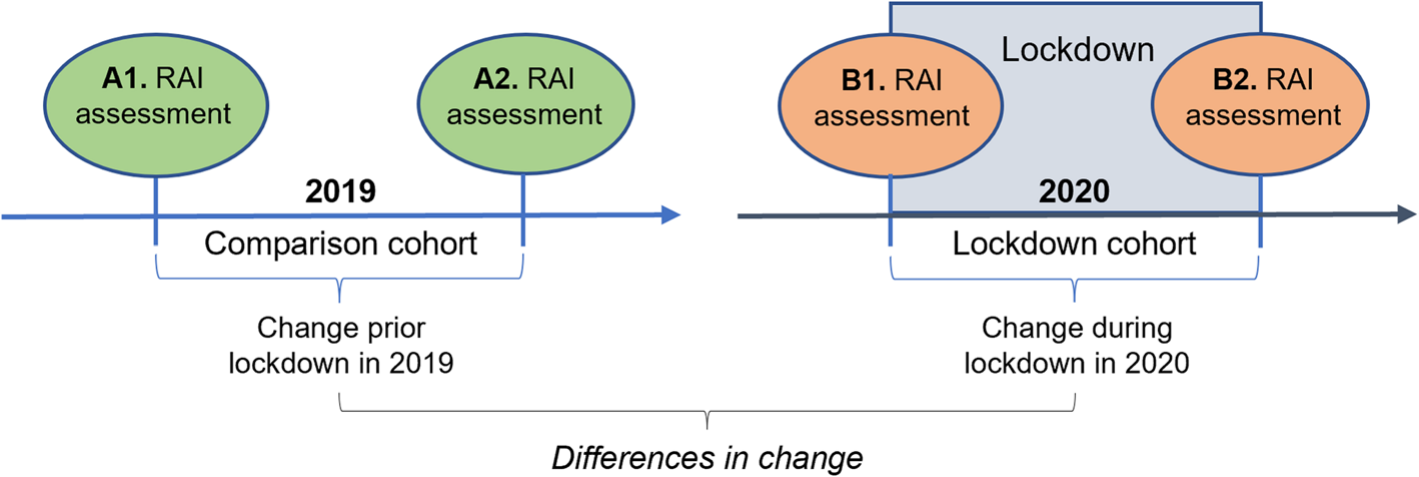
Study design: retrospective register-based cohort study measuring differences in change

Figure 1 illustrates the study design of this register-based retrospective cohort study.

### Participants and data collection

**Fig. 2:**
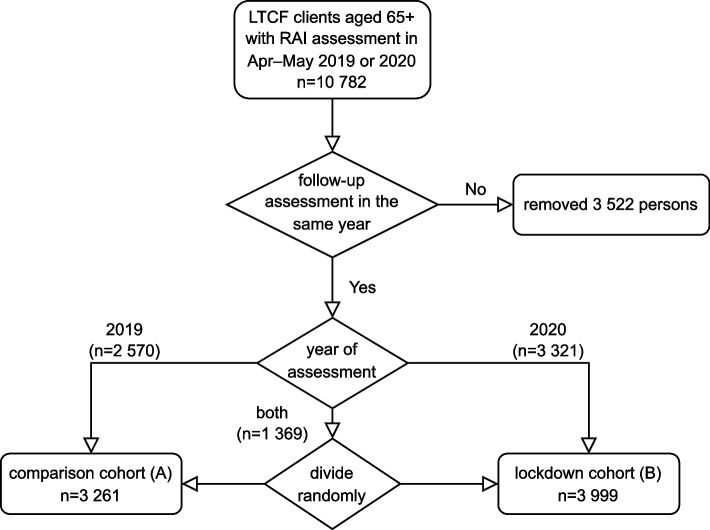
Construction of the study sample and the two cohorts

The inclusion criteria of the study population were Resident Assessment Instrument (RAI) assessed long-term care facility resident aged 65 years old and older with two consecutive RAI assessments in 2019 or 2020 in given time frame: first assessment in April–May 2019 or 2020 and follow-up assessment in the same year at least 90 days later but at its latest on 31st December (*n* = 7 260, Figs. 1 & 2). The RAI instrument is used to evaluate the clinical and functional status and service needs of older people, as well as quality of care [32]. The reliability and validity of RAI instruments have been shown to be good [20, 24] when they are performed by trained nurses following guidelines. Health care professionals, usually trained practical nurses, carry out the RAI assessment twice a year or more often if there are changes in the client’s condition.

The study population comprised two distinct cohorts, measured one year apart, both at two time points (Fig. 1), to compare the difference in the change in the outcome measures between the two cohorts. One of the cohorts was assessed with RAI in the year of the lockdown (2020) and the other in the previous year. The assessments were made at the same time of the year in both cohorts as follows: In *the comparison cohort (A)*, the first (i.e., baseline) assessment occurred on or after 1st April 2019 (A1 in Fig. 1) but not after 31st May 2019, and the next assessment (A2) at least 90 days later but at its latest on 31st December 2019. In *the lockdown cohort (B)*, the first (i.e., baseline) assessment occurred during lockdown (B1), not earlier than 1st April 2020 and not later than 31st May 2020, and the next RAI assessment of the person (B2) at least 90 days later but at its latest on 31st December 2020. Individuals without two consecutive assessments meeting the above criteria were excluded from the study (see Fig. 2). Moreover, persons who would have appeared in both cohorts were randomly assigned to one of them.

The LTCF units were located both in urban and rural areas in Finland. Both public and private units were included. Dates of death were retrieved from the Finnish Digital and Population Data Service Agency and information on confirmed COVID-19 infections from the infectious disease register of the Finnish Institute for Health and Welfare. Register data were linked at the person level using a national personal identifier of the person.

### Study variables

#### Outcomes

We used the following RAI indicators: 1) Changes in Health, End-stage Disease, and Symptoms and Signs scale (CHESS, range 0–5), which indicates health stability, and a higher value indicates greater possibility for hospital or emergency department admission [19], 2) Cognitive Performance Scale (CPS, range 0–6), which assesses the level of cognitive function, with a value of six indicating comatose or not present [34], 3) Activities of Daily Living Hierarchy scale (ADL-H, range 0–6), which assesses performance in the following activities: moving, eating, using the toilet, and taking care of personal hygiene, and a higher value indicates greater difficulties in functioning [33], and 4) Social Engagement Scale (SES, range 0–6), which assesses a resident’s willingness to participate in social opportunities and initiate actions that engage the resident in the life of the nursing home [31]. A higher SES value indicates good social engagement.

#### Adjusting variables

We used age (categorised as 65–74, 75–84 and 85 +), sex (male/female), length of stay in the LTCF (days), dementia diagnosis (yes/no), mortality (1 year from the second assessment, yes/no), baseline value of each outcome at the first assessment, and a comorbidity index as adjusting variables (confounders). Confirmed covid infections was not included as a confounder in the regression models since the number of confirmed infections was so low during the lockdown period (*n* = 14).

For the *comorbidity index* (range 0–10), the clients’ diagnoses according to the ICD-10 classification were first categorised into the following categories: dementia (F00–F03, G30), cancer (C00–C97), diabetes (E10–E14), psychosis, depressive symptoms or other mental health disorders excluding dementia (F04–F99), Parkinson’s disease or other neurological diseases (G00–G99, excluding G30), chronic asthma and chronic obstructive pulmonary disease (COPD) or other respiratory diseases (J00–J99), hip fracture (S72), stroke (I60–I69), ischaemic and other heart diseases excluding rheumatic and alcoholic heart diseases (I20–I25, I30–I425, I427–I52), and other diseases of the circulatory system (I00–I15, I26–I28, I70–I99). Then, the number of manifested classes was calculated for each person.

#### Ethical considerations

The Institutional Review Board (IRB) of the Finnish Institute for Health and Welfare (THL) reviewed and approved the study (11/2011§419-§430), and the research was conducted with permission from the Finnish Institute for Health and Welfare (THL/1118/6.02.00/2021) in accordance with the Finnish legislation (the Act on Data Protection 1050/2018, the Act on the Secondary Use of Health and Social Data 552/2019, and the Act on the Finnish Institute of Health and Welfare 668/2008).

#### Data analysis

*Descriptive statistics* were calculated to explore and compare the characteristics of the comparison and lockdown cohorts. Patient characteristics are expressed as counts and percentages (Table 1). Differences between the samples were examined using independent sample hypothesis testing (chi-squared test and Student’s t test), as well as the standardised mean difference (SMD). Differences were considered negligible if *p* > 0.05 and SMD < 0.1 (for SMD, see e.g., [4]). All the statistical analyses were conducted using R software (version 4.4.0 [39]).

**Table 1:** Baseline characteristics of the comparison and lockdown samples, n (%) or mean (SD)

	Comparison sample; n = 3261	Lockdown sample; *n* = 3999	*p* value (chi-squared or *t* test)	Standardised mean difference (SMD)
Age at baseline, mean (SD)	84.57 (7.5)	83.99 (7.7)	** < 0.001**	0.097
65–74, n (%)	370 (11.3)	564 (14.1)		
75–84, n (%)	1 092 (33.5)	1 389 (34.7)		
85 + , n (%)	1 799 (55.2)	2 046 (51.2)		
Sex, Female, n (%)	2 364 (72.5)	2 809 (70.2)	**0.037**	0.050
Length of stay at baseline, years, mean (SD)	2.77 (3.0)	2.66 (3.1)	0.096	0.039
Comorbidity index, mean (SD)	2.08 (1.6)	2.10 (1.6)	0.453	0.018
Diagnosis of Alzheimer’s disease and related dementias, n (%)	2 496 (76.6)	3 021 (75.6)	0.350	0.023
Depressive mood (DRS = 3–14), %	961 (29.5)	1 168 (29.2)	0.820	0.006
Died within one year after follow-up, %	1 093 (33.5)	935 (23.4)	** < 0.001**	0.226
Health stability (CHESS), %			0.098	0.051
0 (No instability)	1 090 (33.4)	1 271 (31.8)		
1 (Mild instability)	905 (27.8)	1 078 (27.0)		
2–5 (Moderate to severe instability)	1 264 (38.8)	1 649 (41.2)		
Cognition (CPS), %			0.643	0.022
0‒2 (No to mild impairment)	958 (29.4)	1 165 (29.1)		
3 (Moderate impairment)	1 183 (36.3)	1 492 (37.3)		
4–6 (Increased to severe impairment)	1 120 (34.3)	1 342 (33.6)		
Activities of daily living (ADL-H), %			0.657	0.022
0–2 (No to mild impairment)	1 006 (30.8)	1 266 (31.7)		
3–4 (Moderate impairment)	1222 (37.5)	1 460 (36.5)		
5–6 (Severe impairment)	1 033 (31.7)	1 273 (31.8)		
Social Engagement Scale (SES), %			0.225	0.046
0–1 (Low social engagement)	1 028 (41.8)	1 432 (42.5)		
2–3 (Moderate social engagement)	699 (28.4)	1 002 (29.8)		
4–6 (Good social engagement)	730 (29.7)	933 (27.7)		
Distance between assessment dates, mean (SD)	177.4 (28.3)	180.3 (28.0)	** < 0.001**	0.103
CHESS change, mean (SD)	0.22 (1.1)	0.24 (1.1)	0.395	0.020
CPS change, mean (SD)	0.21 (0.7)	0.20 (0.7)	0.720	0.008
ADL-H change, mean (SD)	0.29 (0.9)	0.29 (0.9)	0.942	0.002
DRS change, mean (SD)	0.14 (1.4)	0.14 (1.3)	0.823	0.005
SES change, mean (SD)	−0.18 (0.9)	−0.21 (0.9)	0.373	0.024

*ADL-H* Activities of Daily Living Hierarchy, *CHESS* Changes in Health, End-Stage Disease and Symptoms, *CPS* Cognitive Performance Scale, *DRS* Depression Rating Scale, *SES* Social Engagement Scale

*Survivor bias* originating from the requirement to survive until the second assessment was investigated by calculating additional descriptive statistics based on a larger sample containing all available first assessments, regardless of whether there was a follow-up assessment.

*Logistic regression analyses* were performed to determine whether belonging to the lockdown cohort (predictor) was associated with worsening (of at least 1 unit) of the CHESS, CPS, ADL-H, or SES variable (outcomes). As adjusting variables, we used age, sex, length of stay in the LTCF, dementia diagnosis, mortality, baseline value of each outcome, and comorbidity, as described above. For each outcome variable, we removed persons with missing values (CHESS: *n* = 13, CPS: *n* = 7, ADL-H: *n* = 7, SES: *n* = 1 485) and “worst” values (that is, scale value that describes the weakest possible state) at the baseline assessment so that “worsening” of the outcome would make sense. The worst values were defined as 4 and 5 for CHESS, 6 for CPS, 6 for ADL-H, and 0 for SES. The association was considered statistically significant if 1 was not included in the 95% confidence interval of the odds ratio.

Additional *subgroup analyses* were performed to explore the cohort effect among 1) the oldest age group (85 years old and older at the first assessment), 2) the most severely cognitively impaired (dementia diagnosis and CPS score 4–6 at the first assessment), and 3) those who experienced the lowest social engagement (SES score 0–1 at the first assessment).

In addition, we performed a *sensitivity analysis* to determine whether the distance in days between the first and second assessments influenced the outcome. First, we explored the distribution of the distance. Second, we explored whether distance had a linear effect on the change in each outcome variable. Because distance did not seem to affect any of the outcome variables, we did not include it as an adjusting variable in the regression models.

## Results

### Baseline characteristics

There were nonnegligible differences between the cohorts in any of the outcome measures, that is, health stability, cognition, coping with activities of daily living, or social engagement measured at the first assessment (see Table 1). At the first assessment in both cohorts, the average length of stay in LTCF was three years, the residents had an average of two comorbidities, and 30% of them had a depressive mood. Two-thirds of the residents had unstable health, and similar percentages suffered at least from moderate cognitive impairment or moderate ADL impairment. In addition, approximately 40% of the residents exhibited low social engagement.

There were some differences between the cohorts regarding other than outcome variables. The size of the lockdown cohort was greater (*n* = 3 999) than that of the comparison cohort (*n* = 3 261), and there was a slightly smaller percentage of women (*n* = 2 809, 70%) than in the comparison cohort (*n* = 2 364, 73%). Additionally, the residents of the lockdown sample were on average younger (M = 83.99, SD = 7.70) than those in the comparison sample (M = 84.57, SD = 7.46), and fewer clients died within a year of the second assessment (*n* = 935, 23%) compared to those in the comparison sample (*n* = 1 093, 34%). Additionally, residents in the lockdown cohort had more days between the assessments (M = 180.3, SD = 27.96) than did those in the comparison cohort (M = 177.4, SD = 28.29).

### Survivor bias

When investigating baseline characteristics without the requirement of having survived until the follow-up assessment (cohort A: n = 5 093, cohort B: n = 6 176, data not shown), some age differences remained between study samples, so this is likely not caused by our sampling procedure but rather by regular yearly fluctuations. On the other hand, the difference in gender representation vanished. Additionally, there was no difference in one-year mortality calculated from the first assessment between the samples. Thus, survivor bias likely affects sex distribution as well as mortality. Curiously, there was less social engagement in the lockdown year when considering only the first assessments. This is also suggested in Table 1, where “good social engagement” at the first assessment was slightly lower in the lockdown cohort, but the overall difference remained negligible.

### Multivariate logistic regression models

Belonging to the lockdown cohort was not significantly associated with a decrease in any of the outcome variables, including health stability (CHESS, OR = 1.06, *p* = 0.25), cognitive performance (CPS, OR = 1.05, *p* = 0.43), coping with activities of daily living (ADL-H, OR = 1.03, *p* = 0.67), or social engagement (SES, OR = 0.91, *p* = 0.41), during the six-month follow-up period (Table 2). The 95% confidence interval is located at approximately 1 and very narrow for all other outcome variables except SES, for which it is slightly wider (95% CI [0.73, 1.14]). This indicates a high confidence that the cohort had an extremely small effect on these three variables.

**Table 2:** Multivariate regression models. Statistically significant values are shown in bold

	CHESS decline	CPS decline	ADL-H decline	SES decline
	*n* = 6861	*n* = 6541	*n* = 6343	*n* = 4332
Characteristics	OR (95% CI)	OR (95% CI)	OR (95% CI)	OR (95% CI)
Lockdown cohort (REF = comparison cohort)	1.06 (0.96–1.18)	1.05 (0.93–1.20)	1.03 (0.91–1.15)	0.91 (0.73–1.14)
Value of the scale at baseline	**0.64 (0.61–0.68)**	**0.77 (0.73–0.81)**	**0.81 (0.78–0.84)**	**0.74 (0.69–0.80)**
Age at baseline				
65–74	REF	REF	REF	REF
75–84	**1.23 (1.03–1.47)**	1.03 (0.84–1.28)	1.04 (0.86–1.27)	0.87 (0.62–1.23)
85 +	**1.20 (1.01–1.43)**	0.97 (0.79–1.20)	0.95 (0.78–1.15)	0.79 (0.57–1.11)
Gender (REF = female)	1.09 (0.97–1.22)	0.98 (0.85–1.13)	1.02 (0.90–1.17)	1.19 (0.93–1.53)
Length of stay at baseline	**0.92 (0.90–0.93)**	**0.98 (0.95–1.00)**	0.98 (0.96–1.00)	**0.87 (0.82–0.92)**
Comorbidity index, two or more morbidities	**1.13 (1.02–1.26)**	**1.16 (1.02–1.33)**	1.03 (0.92–1.17)	**0.78 (0.63–0.97)**
Alzheimer’s disease and related dementias	**1.19 (1.05–1.35)**	1.52 (1.30–1.80)	**1.60 (1.39–1.85)**	**0.74 (0.59–0.95)**
Died within one year after follow-up	1.78 (1.58–2.00)	1.99 (1.73–2.28)	**2.20 (1.94–2.51)**	**0.59 (0.44–0.78)**

*ADL-H* Activities of Daily Living Hierarchy, *CHESS* Changes in Health, End-Stage Disease and Symptoms, *CI* confidence interval, *CPS* Cognitive Performance Scale, *REF* reference, *SES* Social Engagement Scale

A low baseline score on any of these outcome scales was associated with a greater increase in the score, which, on most of the scales, indicates a decrease during follow-up. For instance, a low cognitive performance score (CPS) at baseline was associated with a greater probability of an increase in that score during follow-up (OR = 0.03, *p* < 0.001), signifying a decrease in cognitive performance. Note that the SES is reversed compared to other outcomes of the study; that is, a higher score indicates increased social engagement. Accordingly, a low SES (i.e., poor social engagement) at baseline was associated with a minor decrease in SES during follow-up (OR = 0.04, *p* < 0.001)*.* However, there were no statistically significant differences between the comparison and lockdown cohorts.

*Subgroup analyses* (Online Resource: Supplementary Tables 1–3) indicated that in the oldest age category (85 + , cohort A: *n* = 1 799, cohort B: *n* = 2 046), the decline in health stability was somewhat greater in the lockdown cohort than in the comparison cohort (OR = 1.16, p = 0.05, 95% CI [1.00, 1.34]). That is, the oldest persons in the lockdown cohort experienced a greater decline in health stability during the follow-up period than did those of similar age in the comparison cohort. Additionally, in the most severely cognitively impaired subgroup (diagnosis of dementia or CPS 4–6, cohort A: *n* = 993, cohort B: *n* = 1 173), the decline in cognitive performance was greater in the lockdown cohort than in the comparison cohort (OR = 1.37, *p* = 0.03, 95% CI [1.04, 1.82]). There was no statistically significant cohort effect in the low social engagement subgroup (SES 0–1, cohort A: *n* = 1 028, cohort B: *n* = 1 432).

## Discussion

According to this register-based study, COVID-19 lockdown was not associated with deterioration of health stability, cognitive performance, coping with activities of daily living (ADL), or social engagement among 65-year-old and older LTCF residents in Finland. However, our subgroup analysis revealed that among the most cognitively impaired residents, lockdown was associated with increased cognitive decline. Moreover, among the oldest residents aged 85 years old and older, lockdown was associated with decreased health stability. Accordingly, the result shows that lockdown could have affected people in different ways depending on their individual characteristics in the begin of the lockdown period as the customer base in LTCF includes a wide variety of customers regarding for instance their age, health status, physical functioning, cognition, and social network. To our knowledge, this is the first quantitative study among this vulnerable group of older people with an extensive register-based database covering nearly half of the LTCF population in Finland addressing the impacts of the COVID-19 lockdown in Finnish LTC facilities.

Earlier research findings on the effects of COVID-19 lockdown on LTCF residents have been contradictory. A recent review by Benzinger et al. [7] summarising 62 full-text articles revealed that studies examining cognitive and functional decline were limited in number and presented mixed findings. For instance, Canadian and Dutch cohort studies utilising similar register-based RAI data as our study did not find clinically relevant negative effects of the lockdown on mood, behaviour, delirium [3, 27], behavioural problems, or social and cognitive functioning [27]. However, opposite results also exist, suggesting that after the lockdown period, cognition and functioning were lower [5, 35, 37] in LTCF residents.

Our findings on the maintenance of *ADL functioning* among LTCF residents during COVID-19 lockdown is supported by several other studies using routine data (*n* = 5, [6]), direct assessment [16], as well as interviews and surveys [23] suggesting that despite social distancing due to lockdown the LTCF residents were active enough to maintain their existing level of physical and ADL functioning. However, some previous studies have demonstrated a decline in physical or ADL functioning, as confirmed by routine or assessment based [36],  [9] or qualitative [35] data. In these cases, detecting decline in functioning of the residents, lockdown may have accelerated the functional decline that was already present.

In this study, we did not find statistically significant differences in the *social engagement* of residents due to the lockdown. Several studies support our findings (e.g., [3]): Some older people have explained that because they have experienced many crises during their life, the COVID-19 epidemic was not such a large shock for them [1]. For instance, some people had already experienced a pandemic that happened a long time ago. These older people were probably able to confront things “as they were”. They felt that the lockdown was a necessary measure that might have facilitated enduring the situation in terms of resilience [2]. This finding challenges the insights of some researchers stating that the measures taken to protect residents’ health due to COVID-19 were short-sighted in terms of the social dimension of well-being, as residents experienced anxiety, grief, and severe stress, possibly due to a lack of social contact [35]. There are also contradictory results suggesting that the lockdown strongly affected the social wellbeing of LTCF residents [6, 43]. When interpreting the results, it should be considered that populations and nationalities are unique in their nature and culture. Thus, especially the need for social interaction may vary greatly for instance between northern and southern Europeans.

Based on our subgroup analyses, the *health stability* of the oldest age group (aged 85 years old and older) decreased more in the lockdown cohort during the COVID-19 lockdown than in the comparison cohort even though people in the lockdown cohort were on average younger than those in the comparison cohort and older age is typically strongly associated with multimorbidity and unstable health [26]. Our finding is in line with earlier studies: the oldest and the frailest people were often the ones who suffered the most from the exceptional restrictions due to COVID-19 [22]. Additionally, due to the COVID-19 epidemic, nonurgent hospital treatments were cancelled and postponed to secure health care resources, which caused congestion of health care services and caused unmet care needs among all age groups [8, 21]. However, the number of confirmed COVID-19 infections was minor in the study population, and mortality rates were stable in LTCF during the lockdown period [8, 21], which suggests that the lockdown procedure was effective in protecting residents from COVID-19 infection and related deaths in LTCF in 2020.

Only in the most severely cognitively impaired residents experienced *cognitive decline* during lockdown. Some studies (e.g., [36]) support our findings on the stability of cognition during lockdown among LTCF residents. However, also concerns about accelerated cognitive decline due to COVID-19 lockdown have been expressed in several quantitative longitudinal studies using interviews and surveys [16–18, 25]  as well as in a qualitative study on Finnish LTCF residents [35]. Some researchers have suggested that the COVID-19 lockdown affected most the wellbeing of LTCF residents without cognitive impairment and was characterised by loneliness and depression (Van der Roest 2020). However, in our study, health, social engagement, and functioning did not decline significantly even in the most cognitively impaired subgroup.

In future epidemics, policymakers and stakeholders should consider whether it is ethical and constitutional to “protect” some frail population groups by restricting their basic rights or whether people themselves may express their own wishes about the level they want to be protected. Authorities as well as social and healthcare units should prepare for future epidemics in such a way that they are better able to combine individual freedom as well as life and health protection aspects. There is a risk that some of the pandemic-era restrictions remain in use at the grassroots level as a common means of fighting infections, although not all restrictions have a legal basis [40] & [41]. Moreover, the differences between LTCF residents’ individual characteristics should be noted when considering future crisis plans. In other words, actions can have different effects depending on the person’s condition.

It is paramount, that the older residents’ wish to experience the physical closeness of their loved ones is respected despite the possible risk of infection even in very old age or presence of dementia or cognitive decline. It should be considered whether it is reasonable or humane to prevent relatives and friends from meeting, for example, a very old and cognitively impaired LTCF resident for fear of infection. The awareness of the proximity of death is often present to frail and old LTC residents anyhow. Thus, even if the hypothetic infection would result in death, it would on many occasions be less devastating than a situation when older resident would be separated from the loved ones during the last days of one’s life. If the resident is not able to express his/her will because of, for instance, a cognitive decline, their relatives, informal care givers and helpers, as well as friends should be listened and acknowledged as they can act as a voice for someone who is unable to communicate independently.

### Strengths and limitations

This study utilised register-based RAI data, which is a valuable and unique source of information on LTCF residents and has not previously been used in Finland to monitor the possible effects following the COVID-19 lockdown. Indeed, it has been difficult to obtain LTCF data during the COVID-19 lockdown due to extensive restrictions, including social distancing. Researchers, such as researchers, were not able to monitor the situation in residential care units; thus, research on the effects of the COVID-19 lockdown in the Finnish LTCF population has been very limited. The results of this study are also applicable outside of Finland and other Nordic countries since the COVID-19 lockdown influenced the lives of older LTCF residents worldwide. Moreover, the social and health care systems as well as populations in Nordic countries are quite similar. An undeniable strength is the study's cohort design, which enables the comparison of the difference in changes in selected outcomes among LTCF residents who lived during the COVID-19 lockdown to those in an earlier similar cohort.

We detected some survivor bias due to sampling since we restricted the study population to include only LTCF residents who had two consecutive assessments; thus, only those who survived until the follow-up assessment were included in the cohorts. In addition, survivor bias likely affected the gender representation and mortality differences, as the residents in the lockdown cohort were younger, there was a greater proportion of males, and fewer clients died within a year of follow-up compared to those in the comparison cohort. Additionally, social engagement was lower at baseline in the lockdown cohort than in the comparison cohort. The increased mortality of the comparison cohort may be caused by the COVID-19 outbreak that occurred in the following year. However, mortality differences between cohorts were at least partly due to survivor bias, as there was no difference in one-year mortality in the survivor analysis, that is, when residents surviving to the next RAI assessment were not required for sampling. The lower social engagement in the lockdown cohort at baseline was also quite natural, as this was the time of COVID-19 restrictions before social distancing became the “new normal” in LTCFs.

### Reliability and validity

The reliability and validity of RAI instruments and assessments have been shown to be good [20, 24] when carried out according to guidelines of the RAI manual. The electronic point by point manual is integrated with the electronic assessment form. Moreover, in Finland, the RAI instrument has been used since 2000, and nowadays most nurses are specifically trained to use the instrument. Typically, a responsible nurse who knows the LTCF resident well carries out the RAI assessment which improves the quality of the data. In 2019, 43% of LTCF residents aged 75 years old and older, and in 2020, 48% were assessed with RAI [12]. RAI assessments should always be carried out together with the residents and their relatives or other informal helper. If the resident himself/herself is unable to communicate verbally there are several non-verbal communication options to be used such as writing, using figures, music, drawings, or proxy respondents’ knowledge. The use of RAI was voluntary and optional in the care services of older people in Finland until April 2023. Since then, RAI has become mandatory (980/2012, 15 a §) when assessing the service needs of older people receiving regular care services as well as in care management.

## Conclusions

Although several earlier research results suggested that social isolation, specifically a lack of social interaction with friends and social participation, may affect a wide range of health and well-being among older adults, the Finnish older people living in LTC facilities survived without drastic outcomes when assessing 65-year-old and older residents. Only those who were the most cognitively impaired, and the residents who were 85 years old and older declined in health stability. This can be considered a sign of resilience among Finnish older people who have survived through wars as well as earlier pandemics. Moreover, we may conclude that older people and society were able to accept and adopt quite draconian restrictions during the exceptional circumstances due to COVID-19 epidemic. Despite this, there has undoubtedly been much suffering at individual level. It would be humane to allow relatives and piers to visits their loved ones who live in LTCF in the end of their life, even in exceptional circumstances when unknown virus is causing an epidemic.

## Supplementary Information

Supplementary Material 1.

Supplementary Material 2.

Supplementary Material 3.

## Data Availability

This register-based study utilised highly sensitive health and social care data which cannot be opened for public. The study was conducted upon a designated research permit (THL/1118/6.02.00/2021) given by the Finnish Institute for Health and Welfare (THL). Even though the data are pseudonymized, individuals can still be distinguished, i.e., pseudonymized data are still personal data, and data protection regulations such as EU’s general data protection regulation (2016/679) and the Finnish Act on Data Protection (2018/1050), must be applied to data processing and sharing.

## Abbreviations

ADL: Activities of daily living
ADL-H: Activities of Daily Living Hierarchy
CHESS: Changes in Health End-Stage Disease and Symptoms
CI: Confidence interval
COPD: Chronic asthma and chronic obstructive pulmonary disease
CPS: Cognitive Performance Scale
DRS: Depression Rating Scale
IRB: The Institutional Review Board
LTC: Long-term care
LTCF: Long-term care facility
M: Mean
n: Number
N/A: Not available
OR: Odds ratio
RAI: Resident Assessment Instrument
REF: Reference
SES: Social Engagement Scale
SD: Standard deviation
SMD: Standardised mean difference
THL: Finnish Institute for Health and Welfare
