# Supplementary material for: Health, functioning and social engagement among older people living in long-term care facilities during the COVID-19 lockdown in Finland: a register-based cohort study
Source: BMC Public Health. 2025 Mar 8;25:929. doi: 10.1186/s12889-025-22032-8 (PMC11890530; doi:10.1186/s12889-025-22032-8)
Supplement: Supplementary file 1 — Supplementary Material 1. [file 12889_2025_22032_MOESM1_ESM.docx]

Health, functioning and social engagement among older people living in long-term care facilities during the COVID-19 lockdown in Finland: A register-based cohort study

*Submitted: BMC Public Health*

Johanna Edgren^1^, Jokke Häsä ^1^, Mari Aaltonen^1,2^

^1^Finnish Institute for Health and Welfare, Finland

^2^Cerontology Research Center, Tampere University, Finland

e-mail address of the corresponding author: [johanna.edgren@thl.fi](mailto:johanna.edgren@thl.fi)

Supplementary table 1. Multivariate logistic regression models for the oldest age category (85+ at baseline). Statistically significant values are bolded.

|  | CHESS | CPS | ADL-H | SES |
| --- | --- | --- | --- | --- |
|  | n=3604 | n=3493 | n=3389 | n=2320 |
| Characteristics | OR (95% CI) | OR (95% CI) | OR (95% CI) | OR (95% CI) |
| Lockdown cohort (REF = comparison cohort) | **1.16 (1.00–1.34)** | 1.14 (0.96–1.36) | 1.06 (0.91–1.25) | 0.84 (0.61–1.15) |
| Value of the scale at baseline | **0.64 (0.59–0.69)** | **0.72 (0.67–0.77)** | **0.80 (0.76–0.84)** | **0.73 (0.65–0.81)** |
| Gender (REF = female) | 1.08 (0.91–1.29) | 0.95 (0.78–1.17) | 0.94 (0.78–1.14) | 1.10 (0.76–1.63) |
| Length of stay at baseline | **0.94 (0.91–0.96)** | 1.01 (0.98–1.04) | 1.00 (0.98–1.03) | **0.78 (0.70–0.86)** |
| Comorbidity index, two or more morbidities | 1.08 (0.93–1.25) | **1.21 (1.01–1.45)** | 0.99 (0.84–1.17) | **0.74 (0.54–1.01)** |
| Alzheimer’s disease and related dementias | 1.05 (0.88–1.25) | **1.55 (1.24–1.96)** | **1.43 (1.17–1.76)** | 0.81 (0.57–1.17) |
| Died within one year after follow-up | **1.55 (1.33–1.80)** | **1.96 (1.65–2.34)** | **1.97 (1.66–2.32)** | **0.66 (0.46–0.94)** |

ADL-H = Activities of Daily Living Hierarchy, CHESS = Changes in Health, End-Stage Disease and Symptoms, CPS = Cognitive Performance Scale, OR = odds ratio, CI = confidence interval, SES = Social Engagement Scale
