# Supplementary material for: Health, functioning and social engagement among older people living in long-term care facilities during the COVID-19 lockdown in Finland: a register-based cohort study
Source: BMC Public Health. 2025 Mar 8;25:929. doi: 10.1186/s12889-025-22032-8 (PMC11890530; doi:10.1186/s12889-025-22032-8)
Supplement: Supplementary file 2 — Supplementary Material 2. [file 12889_2025_22032_MOESM2_ESM.docx]

Health, functioning and social engagement among older people living in long-term care facilities during the COVID-19 lockdown in Finland: A register-based cohort study

*Submitted: BMC Public Health*

Johanna Edgren^1^, Jokke Häsä ^1^, Mari Aaltonen^1,2^

^1^Finnish Institute for Health and Welfare, Finland

^2^Cerontology Research Center, Tampere University, Finland

e-mail address of the corresponding author: [johanna.edgren@thl.fi](mailto:johanna.edgren@thl.fi)

Supplementary table 2. Multivariate logistic regression for the most severely cognitively impaired (diagnosis of dementia or CPS score 4–6 at baseline). Statistically significant values are bolded.

|  | CHESS | CPS | ADL-H | SES |
| --- | --- | --- | --- | --- |
|  | n=1982 | n=1542 | n=1484 | n=771 |
| Characteristics | OR (95% CI) | OR (95% CI) | OR (95% CI) | OR (95% CI) |
| Lockdown cohort (REF = comparison cohort) | 1.08 (0.88–1.33) | **1.35 (1.03–1.80)** | 0.96 (0.76–1.20) | 1.16 (0.65–2.12) |
| Value of the scale at baseline | **0.69 (0.62–0.76)** | **0.70 (0.53–0.92)** | **0.80 (0.73–0.88)** | 0.81 (0.60–1.06) |
| Age at baseline |  |  |  |  |
| 65–74 | REF | REF | REF | REF |
| 75–84 | **1.40 (1.00–1.97)** | 0.95 (0.61–1.50) | 1.04 (0.72–1.50) | 1.34 (0.55–3.77) |
| 85+ | 1.11 (0.80–1.56) | 0.81 (0.53–1.28) | 0.91 (0.63–1.30) | 1.20 (0.49–3.38) |
| Gender (REF = female) | 1.21 (0.96–1.53) | 1.07 (0.79–1.45) | 0.97 (0.76–1.24) | 1.26 (0.67–2.49) |
| Length of stay at baseline | **0.90 (0.87–0.94)** | 0.99 (0.94–1.04) | 0.99 (0.95–1.04) | **0.85 (0.72–0.98)** |
| Comorbidity index, two or more morbidities | **1.25 (1.02–1.55)** | 1.29 (0.97–1.73) | 1.17 (0.93–1.48) | 0.74 (0.41–1.31) |
| Died within one year after follow-up | **1.57 (1.26–1.94)** | **2.97 (2.25–3.94)** | **2.19 (1.72–2.78)** | 0.77 (0.39–1.47) |

ADL-H = Activities of Daily Living Hierarchy, CHESS = Changes in Health, End-Stage Disease and Symptoms, OR = odds ratio, CI = confidence interval, CPS = Cognitive Performance Scale, REF = reference, SES = Social Engagement Scale
