# Supplementary material for: Health, functioning and social engagement among older people living in long-term care facilities during the COVID-19 lockdown in Finland: a register-based cohort study
Source: BMC Public Health. 2025 Mar 8;25:929. doi: 10.1186/s12889-025-22032-8 (PMC11890530; doi:10.1186/s12889-025-22032-8)
Supplement: Supplementary file 3 — Supplementary Material 3. [file 12889_2025_22032_MOESM3_ESM.docx]

Health, functioning and social engagement among older people living in long-term care facilities during the COVID-19 lockdown in Finland: A register-based cohort study

*Submitted: BMC Public Health*

Johanna Edgren^1^, Jokke Häsä ^1^, Mari Aaltonen^1^

^1^Finnish Institute for Health and Welfare

e-mail address of the corresponding author: [johanna.edgren@thl.fi](mailto:johanna.edgren@thl.fi)

Supplementary table 3. Multivariate logistic regression models for those with low social engagement (SES score 0–1) at baseline. Statistically significant values are bolded.

|  | CHESS | CPS | ADL-H | SES |
| --- | --- | --- | --- | --- |
|  | n=2241 | n=1923 | n=1796 | n=995 |
| Characteristics | OR (95% CI) | OR (95% CI) | OR (95% CI) | OR (95% CI) |
| Lockdown cohort (REF = comparison cohort) | 1.02 (0.85–1.24) | 1.14 (0.90–1.43) | 1.03 (0.83–1.28) | 1.06 (0.70–1.61) |
| Value of the scale at baseline | **0.65 (0.59–0.71)** | **0.79 (0.71–0.87)** | **0.78 (0.72–0.85)** | N/A |
| Age at baseline |  |  |  |  |
| 65–74 | REF | REF | REF | REF |
| 75–84 | **1.34 (1.00–1.66)** | 1.34 (0.95–1.92) | 1.17 (0.85–1.62) | 0.82 (0.46–1.48) |
| 85+ | **1.47 (1.11–1.96)** | 0.93 (0.66–1.34) | 1.12 (0.81–1.54) | 0.80 (0.46–1.44) |
| Gender (REF = female) | 1.08 (0.88–1.32) | 1.08 (0.85–1.38) | 1.02 (0.82–1.28) | 1.23 (0.80–1.94) |
| Length of stay at baseline | **0.91 (0.87–0.94)** | 0.98 (0.94–1.02) | 0.97 (0.94–1.01) | 0.95 (0.88–1.02) |
| Comorbidity index, two or more morbidities | **1.22 (1.01–1.48)** | 1.25 (0.99–1.58) | 1.09 (0.88–1.35) | **0.65 (0.44–0.98)** |
| Alzheimer’s disease and related dementias | 1.14 (0.90–1.44) | **1.71 (1.26–2.34)** | **1.70 (1.30–2.24)** | 0.74 (0.48–1.18) |
| Died within one year after follow-up | **1.72 (1.40–2.10)** | **2.04 (1.61–2.59)** | **2.13 (1.70–2.68)** | 0.68 (0.41–1.09) |

ADL-H= Activities of Daily Living Hierarchy, CHESS = Changes in Health, End-Stage disease and Symptoms, OR = odds ratio, CI = confidence interval, CPS = Cognitive Performance Scale, N/A = not available, REF = reference, SES = Social Engagement Scale
